# Utilisation of public eye care services by the rural community residents in the Capricorn district, Limpopo Province, South Africa

**DOI:** 10.4102/phcfm.v4i1.412

**Published:** 2012-10-05

**Authors:** Mologadi D. Ntsoane, Olalekan A. Oduntan, Benjamin L. Mpolokeng

**Affiliations:** 1 Public Health Department, University of Limpopo, South Africa; 2 School of Health Sciences, University of KwaZulu-Natal, South Africa

## Abstract

**Background:**

Visual impairment and blindness are major health problems worldwide, especially in the rural and remote areas of developing countries. Utilisation of eye care services is essential to reduce the burden of visual impairment and blindness, and it is therefore important that it is monitored.

**Objectives:**

The objectives of this study were to determine the level of utilisation of public eye care services and factors that might have influenced their usage in rural communities, Capricorn district, Limpopo Province, South Africa.

**Method:**

A population-based cross-sectional study design was used. Participants were residents in selected rural villages located within approximately 5 km of six Government hospitals. Following ethical approval and receipt of informed consent, a questionnaire with closed and open-ended questions was used to collect information on the utilisation of eye care services and factors that might influence utilisation. Descriptive statistics and Pearson's Chi-square test were used to analyse and compare the data.

**Results:**

Many (62.7%) of the respondents had used the government eye care services in the past. Over fifty-nine per cent (59.3%) of them were satisfied with the services. Factors reported to influence utilisation (such as monthly income, knowledge of available services and the need for regular eye tests) were positively associated with utilisation of eye care services in this study (*p* < 0.05).

**Conclusion:**

Utilisation of eye care services was relatively good, but varied significantly between sites. An awareness campaign by government and non-governmental organisations about eye care services may increase utilisation amongst rural communities.

## Abstrait

**Le recours aux services de soins oculaires publics par des résidents ruraux du district de Capricorn, dans la province du Limpopo, en Afrique du Sud**

**Contexte:**

La déficience visuelle et la cécité sont des problèmes de santé majeurs dans le monde, en particulier dans les zones rurales et isolées des pays en développement. Le recours aux services de soins oculaires est essentiel pour réduire le fardeau de la déficience visuelle et de la cécité, il est donc important de le contrôler.

**Objectifs:**

Les objectifs de cette étude consistaient à déterminer le niveau d'utilisation des services de soins oculaires et les facteurs pouvant influencer leur utilisation dans les communautés rurales du district du Capricorn dans la province du Limpopo, en Afrique du Sud.

**Méthode:**

Une étude transversale basée sur la population a été utilisée. Les participants étaient des résidents de villages ruraux sélectionnés dans un rayon de 5 km autour de six hôpitaux publics. Suite à une approbation déontologique et à la réception d'un consentement éclairé, un questionnaire composé de questions fermées et ouvertes a été utilisé afin de recueillir des informations sur le recours aux services de soins oculaires et les facteurs susceptibles d'influencer leur utilisation. Des statistiques descriptives et un test Khi-carré de Pearson ont été utilisés afin d'analyser et de comparer les données.

**Résultats:**

Un grand nombre (62.7%) de sondés ont eu recours aux services de soins oculaires publics par le passé. Plus de cinquante neuf pour cent (59.3%) d'entre eux étaient satisfaits des services reçus. Les facteurs rapportés comme influençant le recours à ces soins (tels que le revenu mensuel, la connaissance des services disponibles et la nécessité d'examens oculaires réguliers) étaient positivement associés à l'utilisation des services de soins oculaires dans cette étude (*p* < 0.05).

**Conclusion:**

L'utilisation des services de soins oculaires était relativement bonne, mais variait de façon significative selon les sites. Une campagne de sensibilisation menée par des organisations gouvernementales et non gouvernementale sur les services de soins oculaires pourrait permettre d'augmenter leur utilisation dans les communautés rurales.

## Introduction

Visual impairment and blindness are major public health problems worldwide, especially in the developing countries. Those living in the rural and remote areas of the world are usually of lower socio-economic status, and therefore do not have adequate eye care services due to non-availability, non-accessibility and non-affordability of such services. Where such services are available, there may be several barriers to their use such as lack of knowledge of their availability, affordability, cultural beliefs, et cetera. These may lead to visual impairment and blindness. The utilisation of available eye care services is mandatory for reduction of the burden of visual impairment worldwide. It is therefore essential that utilisation is monitored and barrier factors are eliminated by relevant sectors.

### Setting

This study was carried out in villages within 5 km of government hospitals in the Capricorn district, Limpopo Province, South Africa. The district has a population of approximately 1.2 million people and a large proportion live in the rural areas.^1^ There were 38 villages with 35 831 households in the study area^1^ (5 km around the hospitals). There are seven municipalities in the district and each has a government hospital, six of which provide both ophthalmologic and optometric services. The hospitals are the Polokwane-Mankweng complex, which is situated centrally (tertiary level hospital), in the east are three district hospitals (Seshego, W.F. Knobel and Helen Franz), in the south are Lebowakgomo (regional hospital) and Zebediela (district hospital), and lastly to the north is the Botlokwa district hospital. Mainly ophthalmologists and optometrists offer eye care services. Ophthalmologists provide medical and surgical management of eye diseases and optometrists manage the refractive conditions and refer the patients for ophthalmological services when indicated. All the hospitals offer optometry services except the Helen Franz Hospital. However, only the Polokwane-Mankweng complex (tertiary or teaching hospital) has resident ophthalmologists. These ophthalmologists may visit the regional or district hospitals to offer their services, but patients are often referred from these hospitals to the tertiary hospitals for ophthalmological services.

#### Key focus

This study focuses on the utilisation of eye care services provided by the government to the rural communities living within 5 km from government hospitals and factors influencing utilisation of these services.

#### Background

People living in the rural and remote areas are more likely to have visual impairment and blindness because of non-availability; non-accessibility and non-affordability of eye care services. Also, several factors such as lack of knowledge of available services may act as a barrier to the use of available, accessible and affordable eye care services. It is essential that the available services be utilised to avoid visual impairment and blindness and justify the funds spent on provision of such services. Therefore, utilisation of the services needs to be monitored.

#### Trends

Scarcity of eye care services has been reported in the rural areas of many developing countries such as Jamaica,^2^ Latin America and the Caribbeans,^3^ South Africa,^4^ Nigeria^5^ and India.^6^ Poor accessibility to services has also been reported^2–6^ in these countries. Further, non-affordability^3, 6–8^ has been identified as the main contributor to poor eye care utilisation in many rural communities in Jamaica,^2^ South Africa,^4^ Nigeria,^5^ India^6, 8^ and amongst the Timor-Leste.^9^ However, there are factors that may influence utilisation of available, accessible and affordable eye care services. Lack of knowledge of available care and general attitude towards cataract surgery has been reported as barriers to eye care utilisation in rural India.^10^ A study in the United Kingdom indicated that difficulties in patient-doctor relationship and dissatisfaction due to long waiting lists amongst Indians in the Ealing area of West London contributed to poor utilisation.^11^ Studies in rural India,^6, 12^ Timor-Leste,^9^ and Victoria, Australia^13^ found that a lack of knowledge of the available services and poor knowledge of eye diseases negatively influence eye care utilisation. Also, in America^14^ and India,^15^ demographic profiles such as age and gender were found to influence eye care utilisation, and females were more likely to use eye care services. Furthermore, in India,^15^ the Tehran population in Iran,^16^ amongst older Americans^17^ and elderly Chinese in Taiwan,^18^ levels of education were found to influence eye care service utilisation, as the more educated individuals were more likely to use eye care services. The elderly population are more likely to use eye care services, because they are more prone to eye diseases and the ocular manifestations of systemic diseases. Contrarily, societal attitudes and cultural beliefs have been found to constitute barriers to eye care utilisation.^5, 13, 15, 19, 20^ Good quality of eye care services and consumer satisfaction have also been reported to enhance utilisation of eye care services in a rural India study^6^ and in a Timor-Leste study population.^9^

In South Africa, barriers such as, non-availability, cost, poor accessibility of services, non-affordability, poor knowledge of available services, as well as cultural barriers have been reported to prevent people from using eye care services.^20, 21^ Personal communication with the head of the Public Health Department, Limpopo Province, suggests that the province is providing accessible and affordable eye care services. However, there have been no previous studies that have examined how effectively the services are being used or the satisfaction of the users, especially those living in rural communities where such services are highly needed. This study investigated the utilisation of the government eye care services amongst residents in rural villages within 5 km of government hospitals in the Capricorn district of the Limpopo province. Factors that might influence the utilisation of such services were also investigated.

#### Objectives

The objective of this study was to evaluate the utilisation of the services amongst a rural community population, and to investigate the factors that have influenced utilisation.

#### Contribution to field

No previous study that has evaluated utilisation of eye care services in South Africa could be found in the literature. Prevention of visual impairment and blindness is a key Public Health and Health Promotion endeavour. Monitoring of eye care utilisation is essential for eye care providers to evaluate the worthiness of the substantial budget spent on health care services. Further, monitoring and prevention of factors which act as barriers to eye care utilisation are essential in reducing visual impairment and blindness. This study provides information on the level of utilisation of eye care services amongst rural communities, and factors which influence usage.

## Ethical considerations

Ethical approval for this study was obtained from the MEDUNSA Research Ethics Committee, University of Limpopo (MCREC/H/32/2008: PG). Participants were informed of the purpose of the study and their rights as participants. A consent form was signed by each participant and confidentiality of information was maintained during and after the study.

### Potential benefits and hazards

The findings in this study may be useful to eye care managers in the public service, as they will have empirical evidence of the level of eye care services utilisation amongst those living close to government hospitals. This may prompt them to work on improving utilisation amongst these people as well as those in more remote areas. Such action may enhance utilisation of eye care services, which will reduce the prevalence of visual problems, visual impairment and blindness. The participants were not exposed to any hazard as this was a survey study and questions asked did not pose any potential harm to the respondents.

### Recruitment procedure

Volunteers residing within 5 km radius from each of the government hospitals offering eye care (ophthalmological and or optometric) services were included in the study; children and those living outside the study sites were excluded. The field work (data collection) was done during the week and on weekends between 09:00 and 17:00. In rare cases when nobody was available in the residence visited, such a place was revisited. The head of the household or the most elderly adult present in the house during the visit was invited to participate in the study.

### Informed consent

Those who agreed to participate in the study were given the University of Limpopo consent form. All those invited agreed to participate in the study and signed the form.

### Data protection

Collected data are kept in a securely locked cupboard during and after the study, and will be destroyed by shredding after the report has been published. Data captured in the computer were password protected and will be deleted after report publication.

## Trustworthiness

The data were collected by a researcher and trained field workers who had knowledge of eye care services. The procedures were properly reviewed prior to the data collection. The sample size was high enough to represent the study population. The questionnaire was translated from English into Northern Sotho, the local dialect of the participants, to facilitate understanding of the contents. A pilot study was carried out to ensure that the participants understood the contents of the questionnaire. Participants were encouraged to be frank in their responses.

### Reliability

A pilot study was conducted on two groups of 20 subjects in villages outside the study boundary. The participants were given the questionnaire for completion and re-tested after four weeks. The responses obtained during the two sessions were compared to establish reliability of responses. The questionnaire was modified based on the responses from the pilot study.

### Validity

The design and sampling processes were carefully formulated to minimise the possibility of bias. Also, the study population was carefully defined, with the sample size that represented each village included in the study (Table 1). The result focused only on those living within 5 km of a hospital offering eye care services, to eliminate the problem of inaccessibility to the services. The results cannot be generalised to all rural areas of the Capricorn district as only those close to the hospitals were sampled.

**TABLE 1:** Showing the hospitals, the number of villages within 5 km, the number of households in these villages and the number of households sampled.

Hospitals	Number of villages	Number of households	Sample size per hospital
Polokwane-Mankweng	10	11 244	314
Seshego	5	7821	218
W.F. Knobel	8	3040	96
Lebowakgomo	4	2888	80
Zebediela	5	4128	116
Botlokwa	6	6386	178

**Total**	**38**	**35 507**	**1000**

## Methods

### Subjects and material

One thousand adults living in the rural areas within 5 km of government hospitals offering eye care services were included in the study. As these were rural residents, they were of lower economic status and many might not be educated to the level that would provide good employment and adequate salary. A questionnaire with closed and open-ended questions based on the literature review was developed in English, translated into Northern Sotho (the local dialect) and back translated into English to ensure accuracy. The Northern Sotho version was then used in the study. Only adult participants of both sexes, permanently resident in the study sites and who signed the informed consent form were included. Those visiting were excluded.

### Setting

The study was carried out in the rural villages that are located within 5 km of the six government hospitals which have eye care services in the district. These hospitals were selected because they offer eye care (optometric and/or ophthalmologist) services and the 5 km radius boundary was chosen to minimise the effects of non-accessibility of eye care services and cost of transport for eye care services. Thirty-eight villages were seen in a district map to be within the 5 km radius of the selected six government hospitals. There were 35 507 households in the 38 villages.^1^ The hospitals, the number of villages within the 5 km of each hospital, the number of households within each village and the number of sampled households (Table 1).

### Design

A cross-sectional quantitative population based survey was used to conduct the study. Morgan and Krejcie's table of sample size determination^22^ was used to estimate the sample size (number of households) for the study and this provided an estimated 380 households for a 95% confidence interval and 5% margin of error for the 35 507 households. However, it was decided to include 1000 households because the study was being carried out for a higher degree purpose. It was planned to include one person per household in the study and the number of participants per village was based on the population of each village and ranged from 80 to 314 for the 38 villages (Table 1). The sampling interval per village was calculated and used to select the select the 1000 households (1000 participants) included in the study.

### Procedure

Permission to conduct the study was obtained from the Provincial Department of Health and the local chiefs in the communities where the study was conducted. The questionnaire contained questions on demography, utilisation of eye care services and other factors, which included socio-economic status, knowledge of availability eye care services, need for eye care services, as well as perceptions and satisfaction levels of those who had used the services. These variables have been reported in the literature to influence eye care utilisation. The perception of eye care services was assessed with questions such as: ‘Were you satisfied with the eye care received?’ or ‘Were the services in the hospital speedy?’ – ‘YES or NO – give reason(s) for your answer’. Ratings of eye care services were measured on a Likert-type scale of 1–4, where 1 is poor, 2, 3 and 4 are good, very good and excellent respectively for questions such as ‘How would you rate the quality of eye care services that you received at the hospital?’

The questionnaire was administered to the head of household or the most elderly person present in the house during the visit. To ensure good response rates and afford the opportunity to clarify issues relating to the questionnaire, the researcher and research assistants distributed the questionnaires to the selected houses. Illiterate persons were assisted by the researcher and trained research assistants to complete the questionnaire. In such cases, efforts were made to accurately read each question to the person, and responses were accurately recorded as reported. In rare cases when participants indicated that they could not complete the questionnaires immediately, they were given the questionnaire for completion and it was collected later.

### Data analysis

Data were captured with the Statistical Package for Social Sciences (SPSS) version 15. Descriptive statistics were used to describe data and Pearson's Chi-squared tests of the SPSS were used to establish association between utilisation of eye care services and variables (factors) that may influence it.

## Results

### Socio-demographic profiles of the respondents

**FIGURE 1:**
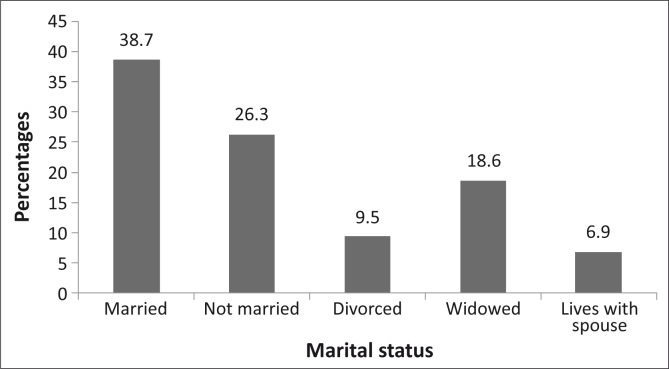
Showing the marital status of the respondents. A few (6.9%) were not married but were living with their spouses.

Eight hundred and fifty-one participants completed and returned the questionnaire, a response rate of 85.1%. The ages of the 841 who reported their ages ranged from 18 to 103 years (mean = 49.2 ± 16.3 years) (*n* = 10 did not give their ages). Respondents (*n* = 843) who indicated their gender included 38.6% male and 61.4% female participants. The marital status, educational status, number of residents per household and monthly salary (Figures 1 – Figures 4). Of the respondents (*n* = 375) who indicated their monthly salary, 77.2% earned three thousand rands or less per month.

**FIGURE 2:**
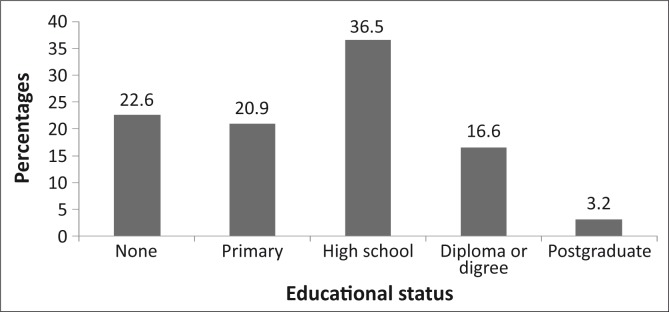
Showing the education status of the respondents. A significant proportion (43.5%) had no education or had primary education.

**FIGURE 3:**
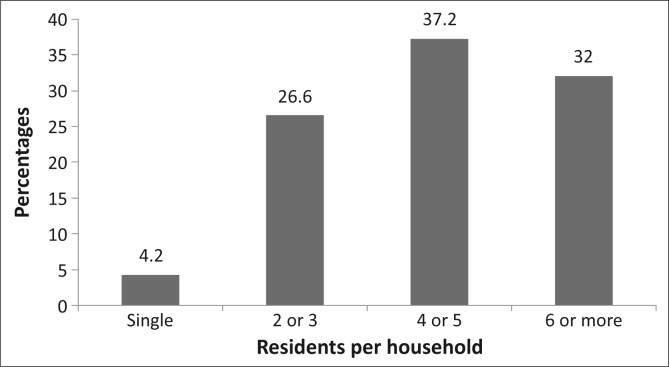
Showing the number and percentages of the residents per household. Nearly seventy per cent (69.2%) of the respondents had 4 or more persons per household.

**FIGURE 4:**
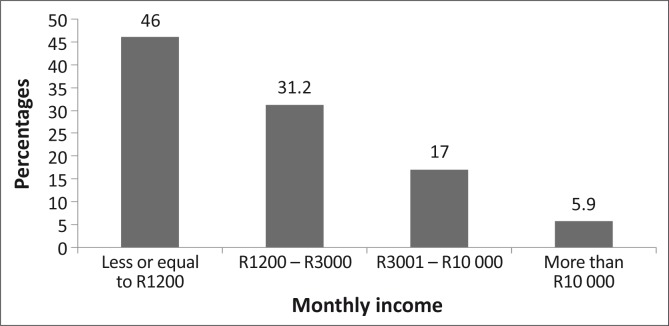
Showing the percentages of those who earned listed ranges of salaries. The majority (77.2%) of the respondents earned R3000 or less per month, which was considered low for the South African standard.

### Knowledge about eye care services

Many (63.4%) of the (*n* = 521) respondents indicated that regular eye examination is important. Of those (*n* = 524) who responded to the consequences of not having regular eye examination, 63.7% reported that serious eye problems could ensue. Of those (*n* = 810) who responded to whether or not children 5 years or younger need eye examination, the majority (79%) indicated that children do need eye examination and the most common reason given was that children might be born with eye problems. The reasons given by those who felt children do not need eye examination included: ‘Children don't have eye problems’, ‘Children are not allowed to test their eyes’ or ‘I didn't know that children need to go for eye tests’. Over half (55.1%) correctly indicated that eye examinations should be done every two years. The majority (78.5%) knew that there were eye care services in government hospitals. Also, 75.5% knew that there were optometrists in government hospitals.

### Need and utilisation of eye care services

In response to whether or not they had experienced eye problems in the past, of the 850 respondents, 69.1% indicated that they had experienced eye problems. The problems reported included poor vision (43.4%), eye diseases (69.1%) and headaches after reading (12.1%). Amongst those (*n* = 847) who responded to whether they were aware that spectacles could be obtained at a cheaper cost at the government hospitals, only 55.1% knew of this. Of the 476 participants who responded to the question on the use of government eye care services, 62.7% had used the services in the past. They included 38% male and 62% female respondents; and of these, 15.5% of males and 27.3% of female respondents had used the services within the past year, and 8.1% of males and 1.1% of female respondents had used the services over five years previously. Knowledge of available services and need for regular eye tests were positively associated with utilisation of services (Pearson's Chi-squared tests, *p* < 0.05). Factors such as age, gender, household size, educational level and occurrence of eye problems were not associated (Pearson's Chi-squared tests *p* > 0.05). In relation to the study sites, percentage utilisation varied significantly amongst the six sites (Pearson's Chi-squared tests, *p* < 0.05). The rate ranged from 23.1% at one of the regional hospitals to 83.3% at the tertiary hospital.

Of the respondents (*n* = 331) who reported having consulted optometrists, 40.2% were males and 58.9% were females; the others did not indicate their gender. Of those (*n* = 331), 36.2% and 63.8% male and female respondents respectively had consulted an optometrist within a year prior to the study. Others had consulted one more than a year prior to the study. Those who had consulted optometrists at the study sites varied significantly (Pearson's Chi-squared tests, *p* < 0.05) and ranged from 38.5% to 90.4%. Just under a third (32.9%) of the respondents had obtained spectacles from the government hospitals. Of the respondents who had consulted an ophthalmologist (*n* = 271), 36.5% had consulted one within the year before this study and included 39% male and 61% female respondents. Others had consulted an ophthalmologist more than one year prior to the study.

Many (59.3%) of the respondents were satisfied with the eye care services received at the hospitals however, the ratings of their satisfaction varied significantly from 38.5% to 90.4% amongst the sites (Pearson's Chi-squared tests, *p* < 0.05). Those who were satisfied gave reasons such as being able to see well with their spectacles and doctors being patient with them. Those who were not satisfied gave reasons such as, long queues, not seeing well with the spectacles received or expecting that spectacles would be provided free of charge. These might have contributed to the poor rating of the eye care services at some of the sites. When asked whether Government hospitals offered good quality eye care services, 68.3% reported ‘yes’. Of the respondents (*n* = 259) who rated the quality of eye care services at the hospitals, 10.8% rated them as excellent, 24.7% rated them very good, 38.6% rated them as good and others (25.9%) rated them as poor. Many (77.1% of male and 72% of female respondents) rated the services between good and excellent.

## Discussions

### Outline of the results

The main objective of this study was to provide information on the level of utilisation of eye care services amongst the rural population living within 5 km of government health care facilities offering eye care services. Also, the relationship between utilisation and factors that have been reported in the literature to influence it were examined. These factors include demography of the respondents, knowledge of available eye care services and the perceptions and levels of satisfaction of those who had used the services.

As the sample population was of low socio-economic status, living in rural villages, with over 43% having no primary school education (Figure 2), and the majority (77.2%) earning R3000 or less per month (Figure 4), they would be expected to benefit from government eye care services. Eye care consultation is free at government hospitals in the province; also, drugs and eye care devices such as spectacles are provided at affordable prices. Compounding their economic status is the fact that people with lower socio-economic status often have large families, as reflected in this study where 32% of the respondents had six or more household members. These socio-economic conditions mean that the majority of this category of people would not have health care insurance, may not be able to afford private eye care services and will have to depend on government eye care services. Therefore, one would expect utilisation in this study sample to be high.

Media reports suggest that the Provincial governments in South Africa are expending a substantial budget in providing health care services (including eye care) to cater for citizens, especially those who cannot afford private services. Hitherto, there has been no information on whether these services are being adequately utilised, especially by the rural communities or whether those for whom they were provided were happy with the services. Therefore, this study provides useful information on these important issues. The fact that many (62.7%) of the respondents had used the public eye care services in the past is considered a good level of utilisation. However, it would be expected that those living further from the hospitals may have lower utilisation rates because of barriers such as non-accessibility and transport costs. It is noteworthy that utilisation varied significantly from one hospital area to another. Therefore, government needs to investigate what might be responsible for the low rates of utilisation of eye care services and poor perception of respondents around some of the hospitals in the district.

There are several factors that may act as barriers to utilisation of eye care services. These include poor knowledge of available services. Several studies have found positive association between good knowledge and greater utilisation of services.^13, 15, 18, 23–25^ In the present study, knowledge of the availability of eye care services and other aspects of eye care were mixed, being good in certain sites, but quite poor in others. However, in general, this study also found an association between knowledge of available eye care services and utilisation (*p* < 0.05). Also, the study revealed a greater utilisation amongst those with a higher monthly income, (*p* < 0.05). However, a previous study by Latinen et al.^26^ did not find an association between economic status and use of services amongst visual impairment patients in government hospital and non-governmental organisations in Finland. This may be due to the differences in the eye and visual status of the subjects, because Latinen et al.^26^ studied visually impaired persons.

Although, factors such as increased age,^11, 12, 14, 24^ female gender,^9, 13, 14, 24^, higher educational level and low household size^14, 16, 17, 25^ have been associated with being more likely to use eye care services, these associations were not found in this study (*p* > 0.05). Furthermore, need such as the presence of eye problems^9, 14^ has been reported to significantly increase utilisation rate of eye care services. In this study, no association was found between occurrence of eye problems and utilisation of eye care (*p* > 0.05). The above imply that utilisation did not increase significantly with age in the present study, in spite of the fact that older persons are more likely to have eye problems and ocular manifestations of systemic diseases, and are therefore more likely to use eye care facilities. This non-association may be due to the disproportionate number of participants in the various age groups of the participants. Also, female and male utilisation levels were not significantly different statistically, presumably due to the higher proportion of female participants in the study, as this will reduce the intra-gender percentage of utilisation. Lack of statistically significant difference in utilisation between those with higher education and those with lower or no education may be due to the higher number of the latter group in the sample population. Although, those with a large household size would be expected to be less likely to use eye care services due to greater family commitments, the non-statistically significant difference in eye care utilisation between those with a large house size and those with a small size in this study may imply that this assumption does not apply to this population, in which most participants were of lower socio-economic status. Further, the lack of statistically significant difference between eye care utilisation amongst those with eye problems, compared to those without, may be attributed to the fact that other means of management such as traditional methods are used by the participants, as this is common amongst rural residents.^20^

Although, consumer dissatisfaction has been considered to be a barrier in sustaining eye care utilisation,^4, 11^ in this study, many respondents (59.3%) were satisfied with the services received at the government eye care facilities and the ratings of their satisfaction was over 90% at one site (*p* < 0.05). This is of interest, as these good satisfaction and ratings would enhance utilisation of services, which would reduce the risks of visual impairment. However, a minority were dissatisfied because of the long queues and long waiting lists. Also, it was noted that satisfaction and ratings differed significantly from one hospital area to the other (*p* < 0.05). This may be related to the differences in quality of services offered; therefore government needs to look into the reasons for such differences.

## Limitations of the study

This is a quantitative study and quantitative studies have their inherent limitations, which may manifest in this study; however, they are recognised to have a key role to play in the development of new knowledge, generating questions and hypotheses that could form the basis for further research.^27^ Another limitation in this study is that participants left some questions unanswered, which led to variation in the number of respondents for the various questions in the study. Furthermore, as the study was carried out amongst those living close to the hospitals, the results cannot be generalised to all those living in the rural areas of the district or province.

## Conclusion

On average, the utilisation of eye care services in this study is considered to be good, being 62.7% amongst the respondents. However, utilisation would be expected to be lower amongst those living father from the hospitals due to cost of transportation. It was noted that the level of utilisation varied significantly between the study sites, suggesting better services in some of the hospitals than others. As knowledge of available eye care services was associated with higher utilisation, this suggests a need for an awareness campaigns on available health care services, especially amongst those who live in the rural and remote areas.
